# A Difficult Problem of Diagnosis

**Published:** 1916-04-08

**Authors:** 


### PROLONGED JAUNDICE.

### A Difficult Problem of Diagnosis.

Jaundice, with few or no other symptoms, is a clinical position which every now and again the practitioner is called on to face. In children and young adults it hardly produces anxiety; such cases are not infrequent, recovery in the course of a few weeks follows some simple form of treatment, and the condition is assumed to be due to catarrhal swelling of the duodenal and biliary mucous membranes. But in adults the position is a less confident one. It is true that here also the catarrhal explanation may apply; and in any individual case the event may show that the diagnosis was justified. But when weeks multiply and develop into months new thoughts necessarily begin to arise; the suggestion of catarrh is felt by the practitioner to be wearing thin, and probably the patient and his friends also display more or less anxiety. The question therefore arises whether some more serious diagnosis is not necessary. "Catarrh" will do very well for a time, but its sustaining virtues are not inexhaustible. If someone could tell us the exact number of weeks or months during which jaundice due to catarrh may continue, he would be a great benefactor. We should know then how long, in the circumstances here contemplated, we were justified in adhering to the possibility of the simpler explanation. It is certain that such jaundice may endure for several weeks, and even for a few months. But it would be idle to pretend that when even the shorter of these two periods is reached the position is not an anxious one. What may be quite true when presented in a general statement is not necessarily true of the individual case upon which a verdict has to be pronounced.

### An Anxious Position.

The question in clinical practice is not, May the jaundice in this patient be due to catarrh? but rather, Is it so due in this particular instance? Exceptional experiences have undoubtedly their measure of helpfulness, but they do not nullify the general rule. Hence it inevitably happens that jaundice in an adult becomes as time advances an increasingly anxious consideration, and that the practitioner who starts cheerfully with a diagnosis of catarrh finds himself ere long more or less perturbed in reference to the accuracy of this diagnosis. And the longer the jaundice lasts the more acute this doubt becomes. A few cases could be quoted in which jaundice has lasted for many months (in one instance nine months, in another eighteen) and has then disappeared. Such cases presumably were examples of chronic catarrh, but their recurrence affords no guarantee that the experience will be repeated, and this especially in view of countless records in which jaundice existing as an isolated clinical observation has proved to be the first event in the evolution of serious organic disease.

At first it may, perhaps, be said that the latter is associated with pain, and that the assumption here being considered is that of jaundice apart from other clinical disturbances. But the first of these two statements requires qualification. It is true that malignant disease of the liver is classed among the "painful" diseases of this organ, but even here there are exceptions, and to obstruct the bile duct a growth need not necessarily be one involving the liver substance. Without question a malignant tumour may exist at the orifice or in the course of the bile duct, may produce a continuing jaundice, and for long may be unaccompanied by pain or by any other symptom. Such a possibility has therefore to be contemplated when jaundice of some weeks or months duration exists in a patient who has reached an age when malignant disease is among the possibilities of the position. The other aspect of this proposition is found in the question, If the jaundice disappears is this a certain proof that the cause of it was not malignant disease? Well, the probabilities in favour of an affirmative reply are certainly very numerous, but they stop short of an absolute conclusion. A patient who has malignant disease may have also catarrhal disturbances in the biliary passages, and this may, of course, disappear under treatment. Hence jaundice might disappear even though malignant disease was present.

### Gall-Stones without Pain.

In reference to gall-stone it is true that attacks of pain are to be expected as features of the clinical history. Yet even here exceptions, though only occasional, have been recognised. Among the records on the subject are one in which after a single attack of "colic" the patient remained jaundiced for six years, and another in which jaundice of nine months' duration, and entirely unattended by either pain, rigors, or vomiting, was cured by the removal of forty-three gall-stones.

There may now be noted one or two observations which, when associated with persisting jaundice, give to this latter a sinister aspect. First is the occurrence of rigors with sharp, short-lived elevations of temperature. To say that these are never met with in catarrhal jaundice might be going too far, but they generally mean organic obstruction. A second threatening feature is evidence of an enlarged gall-bladder. Here again an absolute rule cannot be defined, but such an observation is very much against a diagnosis of catarrh; it leans strongly towards an organic block and favours cancer rather than gall-stone. Such general considerations as loss of flesh and strength have an obvious importance, but they cannot be pushed very far unless, as is rarely the case, they take on an extreme degree. Finally comes the position in which no certain decision seems possible, and where it is therefore necessary to resort to an exploratory incision if the diagnosis is to be confidently secured. It seems just to advise this step if, in spite of thorough medical treatment, jaundice persists, and the physical cause on which it depends cannot be discovered.

